# Implementation barriers and facilitators of digital health interventions for older adults with multimorbidity: a scoping review

**DOI:** 10.3389/fpubh.2026.1896061

**Published:** 2026-07-28

**Authors:** Hai-lian Chen, Shuang Zou, Lin-lin Zheng

**Affiliations:** Nursing Department, The Second Affiliated Hospital, Zhejiang University School of Medicine, Hangzhou, Zhejiang, China

**Keywords:** community health, digital health interventions, facilitators, implementation barriers, multimorbidity, older adults, public health nursing, scoping review

## Abstract

**Objective:**

Although digital health technologies may support chronic disease management, their adoption, integration into care, continued use, and scale-up among older adults with multimorbidity remain difficult in real-world settings. This scoping review aimed to map the types of digital health interventions used for this population, identify implementation barriers and facilitators, and summarize implementation-related support elements that may inform future practice and policy.

**Methods:**

We conducted a scoping review based on the Joanna Briggs Institute methodology and the Arksey and O'Malley framework. Six electronic databases were searched from database inception to 23 June 2026 to locate relevant empirical studies published in English and Chinese.

**Results:**

Twenty articles were included, and implementation-related factors were mapped across five pragmatic domains: individual, technological, care delivery, social, and health system factors. Reported barriers included limited digital literacy, complex interface designs, and fragmented care support. Reported facilitators included age-friendly design, personalized training, and collaborative support networks involving healthcare providers and families.

**Conclusion:**

The implementation of digital health for this population is shaped by a complex interplay of multi-level factors. Future research should prioritize implementation science-based and collaborative models to enhance accessibility and adherence. Our findings may inform the design of more accessible and age-friendly digital care models and support the work of community and public health nurses alongside other care stakeholders. The mapped findings describe reported implementation determinants and should not be interpreted as evidence that any individual function or support element is independently effective.

## 1 Introduction

Population aging has made multimorbidity a major challenge for older adults and health systems (1, 2). Multimorbidity is associated with polypharmacy, competing treatment demands, greater healthcare use and financial burden, and a substantial self-management workload (2–4). Conventional chronic disease management models often rely on single-disease guidance and may not adequately address the complex and individualized needs of older adults with multimorbidity. These challenges highlight the need for integrated, person-centered care tailored to this population.

Digital health technologies—including mobile applications, remote monitoring systems, and artificial intelligence–enabled tools—are increasingly used to support integrated chronic disease management (5). In single-condition populations, such as people with hypertension or heart failure, these technologies have been associated with improved physiological outcomes and quality of life (6). Such findings, however, do not in themselves demonstrate that an intervention can be adopted, incorporated into routine care, sustained by patients and care teams, or delivered at scale in real-world settings. For older adults with multimorbidity, implementation is further shaped by treatment burden, functional limitations, multiple care relationships, and fragmented care pathways. Although the literature on digital health for older adults continues to grow, much of it focuses on single diseases, clinical outcomes, or general technology acceptance rather than the implementation challenges specific to multimorbidity (5, 7, 8). Consequently, the circumstances in which digital health interventions can be feasibly integrated and sustained within continuing care remain poorly understood.

Implementation in this setting is inherently complex. It involves interactions among patients, technologies, family caregivers, healthcare professionals, and healthcare organizations, and the available evidence spans heterogeneous study designs and service contexts (8). Beyond initial uptake, implementation includes usability, integration into care workflows, stakeholder support, continued use, and the potential for broader delivery. This complexity is not readily captured by conventional systematic reviews focused on narrowly defined interventions or outcomes (9). A scoping review is therefore well suited to map the available evidence, identify recurring patterns across care settings, and delineate gaps in knowledge concerning the digital management of older adults with multimorbidity.

Several recent reviews have examined clinical outcomes of digital health interventions for older adults with chronic conditions and barriers to technology adoption in broader older populations with chronic disease (5, 7). However, these reviews have not specifically mapped intervention types, implementation barriers and facilitators, and implementation-related support elements in the context of multimorbidity, where multiple conditions, treatment demands, and care interfaces may create distinctive challenges.

The present review therefore focuses on older adults with multimorbidity as the core population. Caregivers and healthcare professionals were considered implementation stakeholders only when their perspectives, roles, or actions directly informed the delivery, adoption, integration, or continued use of a digital health intervention intended for this population. By bringing together intervention types and functions, reported implementation determinants, and delivery or implementation support elements, this review aims to generate evidence that may inform continuing care, including community and public health nursing practice, multidisciplinary care planning, and policy development. Related reviews have also considered digital health in multimorbidity management, self-management applications, or hospital-to-home transitions; their differing populations and review questions provide complementary rather than directly comparable evidence (10–12).

## 2 Methods

### 2.1 Study design

This scoping review was conducted in accordance with the framework proposed by Arksey and O'Malley (13) and the methodological guidance of the Joanna Briggs Institute (9). The review was reported in accordance with the PRISMA Extension for Scoping Reviews (PRISMA-ScR) (14). A review protocol was not registered. The completed PRISMA-ScR checklist is provided in Supplementary Table S2.

### 2.2 Review questions

The aim of this scoping review was to map the types of digital health interventions and implementation-related evidence for older adults with multimorbidity across continuing-care settings. The review addressed the following questions:

- What types and functions of digital health interventions have been used to support the management of multimorbidity among older adults?
- What barriers and facilitators influence the adoption, integration into care, continued use, or sustainability of these interventions?
- What implementation-related support elements, such as training, technical assistance, caregiver engagement, care coordination, and workflow integration, have been reported or proposed?
- What implications do these findings have for the design and delivery of continuing care, including community and public health nursing practice?

### 2.3 Eligibility criteria

The eligibility criteria for this review were developed according to the Population–Concept–Context (PCC) framework recommended by the Joanna Briggs Institute. The population of interest comprised older adults aged 65 years or above with multimorbidity, defined as the coexistence of two or more chronic conditions. In mixed-population studies, evidence was considered eligible when findings relevant to older adults with multimorbidity could be identified from the report. Formal or informal caregivers and healthcare professionals were not treated as parallel target populations. Studies involving these stakeholders were eligible when their perspectives, roles, or outcomes directly informed the implementation of a digital health intervention intended for older adults with multimorbidity. The concept focused on digital health interventions used in the management of multimorbidity, including but not limited to mobile health applications, remote monitoring systems, wearable devices, digital self-management platforms, telehealth services, and digital care coordination tools. Eligible studies needed to report implementation-relevant barriers, facilitators, processes, or support elements. Implementation-related support elements were defined as resources, strategies, or organizational arrangements intended to facilitate adoption, integration into care, continued use, or potential scale-up, including training, technical support, workflow integration, caregiver engagement, and care coordination. The context included community health services, primary healthcare institutions, outpatient or ambulatory services, home-based care, transitional care, and post-discharge continuing management. Studies with an inpatient component were eligible when the intervention extended into transitional or continuing care; studies limited solely to an acute inpatient episode without a continuing-care function were excluded. Eligible evidence sources included qualitative, quantitative, and mixed-methods studies published in peer-reviewed journals. Conference abstracts, editorials, commentaries, and duplicate publications were excluded. Only studies published in English or Chinese were included in the review.

### 2.4 Search strategy

A three-step search strategy was developed in accordance with Joanna Briggs Institute guidance. First, exploratory searches of PubMed and CINAHL were undertaken to identify relevant literature and refine the search terms used in the final strategy. Second, a comprehensive search was conducted in Web of Science Core Collection, PubMed, CINAHL, Embase, China National Knowledge Infrastructure (CNKI), and Wanfang from database inception to 23 June 2026. Database inception was defined as the earliest searchable or indexed date available in each database. The searches were developed around the review concepts of multimorbidity, older adults, and digital health interventions, with database-specific adaptations. Controlled vocabulary, where available, was combined with free-text terms and adapted to the syntax and indexing features of each database.

In Web of Science Core Collection, PubMed, CINAHL, and Embase, multimorbidity, older adults, and digital health interventions were combined as separate search blocks. To maximize sensitivity in CNKI and Wanfang, the electronic searches combined multimorbidity and digital health intervention terms, while the age criterion was applied during screening. Implementation-related terms, such as implementation, barriers, facilitators, acceptability, adoption, feasibility, and usability, were not used as a mandatory additional AND block because implementation-relevant information is not consistently indexed and may be reported only in the full text. Instead, implementation relevance was assessed during title and abstract screening and confirmed during full-text review. Eligibility with respect to language and publication type was also assessed during screening; only peer-reviewed empirical studies published in English or Chinese were eligible. Full search strategies are provided in Supplementary Table S1.

### 2.5 Study selection

**Figure 1:**
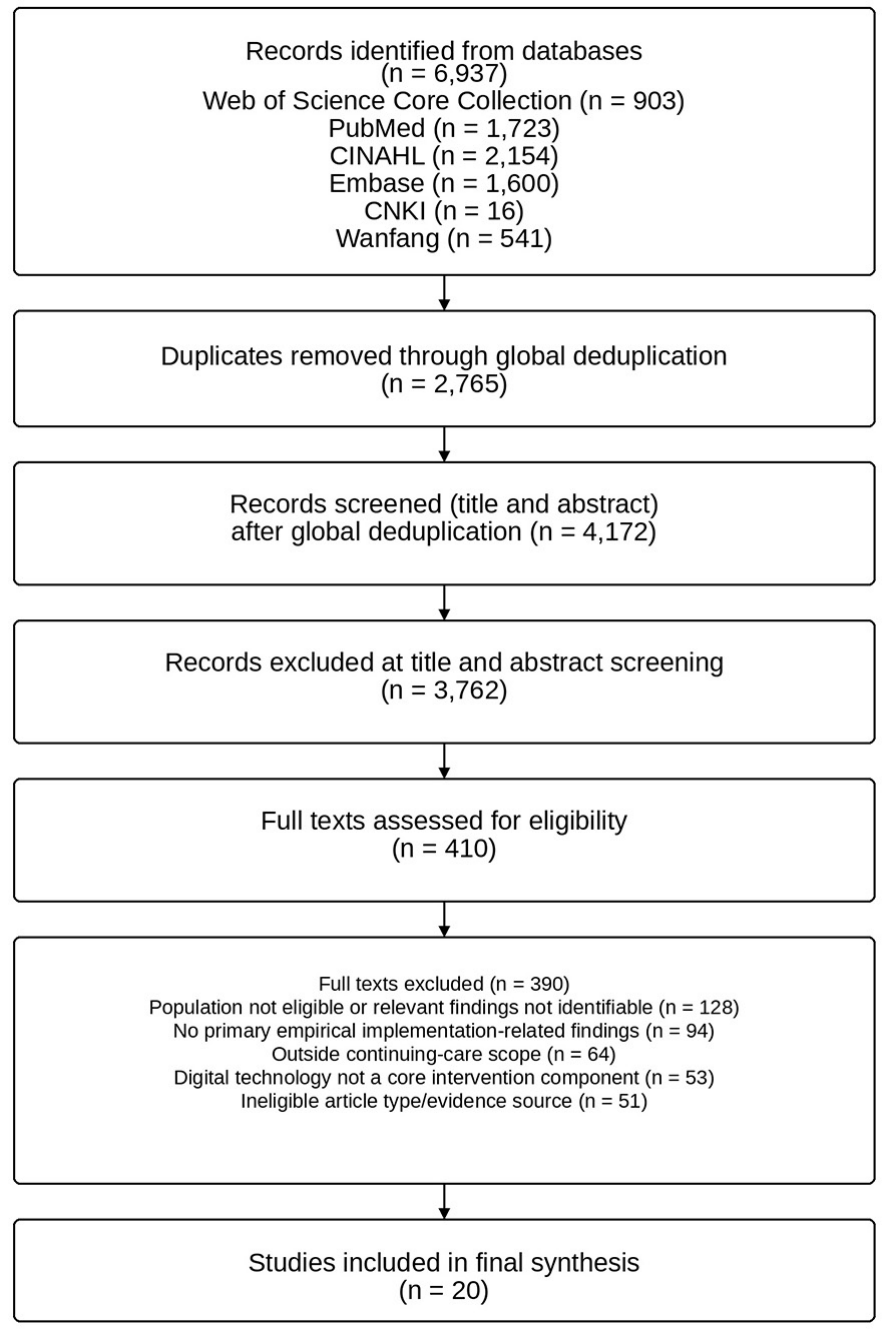
PRISMA-ScR flow diagram of the study selection process.

All retrieved records were imported into EndNote 21, merged, and globally deduplicated before screening. Two reviewers independently screened titles and abstracts and subsequently assessed full texts against the predefined eligibility criteria. Records were not excluded at the title and abstract stage solely because implementation-related terms were absent from the title or abstract. During full-text assessment, eligible studies were required to report primary empirical findings relevant to implementation, including acceptability, usability, feasibility, reach, adoption, actual use or engagement, delivery, workflow integration, sustainability, or barriers and facilitators. Disagreements were resolved through discussion, and a third reviewer was consulted when consensus could not be reached. The study selection process is presented in the PRISMA-ScR flow diagram (Figure 1).

### 2.6 Data extraction

A standardized data-charting form was developed by the research team to collect relevant information from each included study. The charted data included author and year of publication, country or region, study design, target-population characteristics, care context, digital health intervention type and functions, stakeholder involvement, and reported implementation barriers, facilitators, and implementation-related support elements.

For each intervention, we charted reported core digital functions, including monitoring, reminders, education, communication, self-management support, data feedback, and symptom or goal reporting, together with reported delivery or implementation support elements, including age-friendly design, personalization, training, technical support, caregiver involvement, professional follow-up, workflow integration, and care coordination. These descriptors were used to characterize reported intervention features and implementation support, rather than to identify independently effective components.

### 2.7 Data synthesis

Given the exploratory nature of scoping reviews, a descriptive analytical approach was used to synthesize the findings. Data concerning implementation barriers, facilitators, and support elements were first coded from the included studies. The Consolidated Framework for Implementation Research (CFIR) was used as a sensitizing framework to guide interpretation during thematic synthesis, while the five reporting domains—individual, technological, care delivery, social, and health system factors—were retained as pragmatic summaries of the extracted data rather than as one-to-one CFIR constructs. Factors could be considered across more than one domain where appropriate. The findings were summarized using tables, thematic categorization, and narrative synthesis to provide an overview of the current evidence landscape regarding the implementation of digital health interventions for older adults with multimorbidity. Consistent with the methodological guidance from the Joanna Briggs Institute for scoping reviews, formal methodological quality assessment of the included studies was not performed.

Digital health interventions were grouped inductively according to their primary functions and delivery modes. The resulting categories were reviewed against the World Health Organization Classification of Digital Interventions, Services and Applications in Health (15) and retained as pragmatic descriptive groups rather than one-to-one taxonomy categories. CFIR was not used as a search, eligibility, or formal primary coding framework. For reporting purposes, individual factors encompassed health, functional status, cognition, digital skills, beliefs, self-efficacy, and perceived value; technological factors encompassed interface design, usability, reliability, interoperability, devices, and network connectivity; care delivery factors encompassed professional support, training, workload, follow-up, care coordination, and workflow integration; health system factors encompassed organizational resources, policy, reimbursement, institutional processes, and infrastructure; and social factors encompassed support from family, caregivers, and peers, trust, social relationships, and social digital divides. A factor could be assigned to more than one domain. At the study level, each study was counted once within a domain when it reported at least one barrier or facilitator in that domain; domain-level frequencies could therefore overlap and were not summed across domains.

## 3 Results

### 3.1 Study selection

The study selection process is summarized in the PRISMA-ScR flow diagram (Figure 1). The database searches identified 6,937 records, including 903 from Web of Science Core Collection, 1,723 from PubMed, 2,154 from CINAHL, 1,600 from Embase, 16 from CNKI, and 541 from Wanfang. After global deduplication, 4,172 unique records remained for title and abstract screening. Of these, 3,762 records were excluded. Full texts of 410 reports were assessed for eligibility, and 390 reports were excluded. The primary reasons for exclusion were that the target population was not eligible or findings relevant to adults aged 65 years or older with multimorbidity could not be identified from the report (*n* = 128); no primary empirical implementation-relevant findings were reported (*n* = 94); the setting fell outside the continuing-care scope, such as interventions confined to acute inpatient care without ongoing post-discharge management (*n* = 64); digital technology was not a core component of the intervention (*n* = 53); or the article type or evidence source was ineligible, including reviews, study protocols, commentaries, conference abstracts, or non-peer-reviewed sources (*n* = 51). Ultimately, 20 studies met the eligibility criteria and were included in the final synthesis.

### 3.2 Characteristics of included studies

A total of 20 studies published between 2011 and 2025 were included in this review. Most studies were conducted in North America, including 5 from the United States and 4 from Canada. In Europe, studies were reported from Germany (*n* = 2), Spain (*n* = 2), Italy (*n* = 1), and Ireland (*n* = 1). Additional studies were conducted in Asian regions, including China (*n* = 3), South Korea (*n* = 1), and Thailand (*n* = 1). Within the community, outpatient, primary care, and transitional-care scope defined for this review, the included interventions were delivered predominantly in community settings, home environments, or primary healthcare institutions. This distribution should be interpreted in light of the review's eligibility criteria, rather than as evidence of the settings in which digital health services are generally delivered to older adults.

The included studies employed diverse research designs. Qualitative studies were most common (*n* = 8, 40.0%), followed by randomized controlled trials (*n* = 3, 15.0%) and cross-sectional studies (*n* = 3, 15.0%). Mixed-methods studies (*n* = 2, 10.0%) and feasibility trials (*n* = 2, 10.0%) were less frequent, while one longitudinal trial (5.0%) and one co-design study (5.0%) were identified.

Sample sizes ranged from 12 to 752 participants, and participants were generally older than 70 years on average; the highest reported mean age was 79.44 years. Detailed characteristics of the included studies are provided in Table 1.

**Table 1:** Characteristics of included studies.

References	Country/region	Participants	Digital health intervention	Study design
Wakefield et al. (22)	USA	*N* = 302; mean age 68; veterans with diabetes and hypertension	Nurse-managed home telehealth monitoring	Randomized controlled trial
Mira et al. (31)	Spain	*N* = 99; mean age 70.9–72.9; older adults taking multiple medications	Virtual pillbox app (ALICE) with images and reminders	Randomized controlled trial
Donesky et al. (25)	USA	*N* = 15; mean age 71; COPD and heart failure	Home-based videoconferencing yoga (TeleYoga)	Feasibility study
Bernocchi et al. (24)	Italy	*N* = 112; mean age 70; severe combined COPD and chronic heart failure	Integrated telerehabilitation with nurse calls	Randomized controlled trial
Portz et al. (28)	USA	*N* = 24; mean age 78.4; multiple chronic conditions	Patient portal (My Health Manager)	Qualitative study
Schmidt et al. (23)	Germany	*N* = 40; mean age 73.7–77; living alone with about 2.8 chronic diseases	Care management and video conferences via tablets	Qualitative study
Ha and Park (21)	South Korea	*N* = 226; mean age 79.44; two or more chronic diseases	ICT-based healthcare and wearable device monitoring/acceptance	Cross-sectional descriptive study
Scheibe et al. (17)	Germany	*N* = 12; mean age 78.7; multimorbidity and mild cognitive impairment	Telemonitoring app (Motiva) for vital data	Formative qualitative evaluation
Hall et al. (30)	Canada	*N* = 14; mean age 73.8; cancer and multimorbidity	Technology-supported self-management and information seeking	Qualitative descriptive study
Steele Gray et al. (34)	Canada	*N* = 45; mean age 70.2; COPD and cancer	Electronic patient-reported outcome tool for goal elicitation and modification	Mixed-methods trial
Kuo et al. (33)	Taiwan, China	*N* = 20; mean age 72.8; community older adults with chronic disease	Digital management concept using photo diaries	Qualitative study
Tahsin et al. (35)	Canada	*N* = 22; mean age 75.1; mean 4.88 chronic conditions	Goal-supporting ePRO mHealth app and portal	Descriptive qualitative study
Sheng et al. (16)	Ireland	*N* = 60; mean age 74; heart disease, COPD, and heart failure	ProACT digital platform for symptom and well-being monitoring	Longitudinal trial
Nambisan et al. (19)	USA	Age 60–80; multiple chronic conditions	myHESTIA comprehensive digital self-care support system	Mixed-methods study
Xu et al. (29)	Hong Kong, China	*N* = 752; mean age 68.3; two or more chronic conditions	Online health services and social media	Cross-sectional study
Buawangpong et al. (26)	Thailand	*N* = 29; mean age 71; multimorbidity	Telemedicine through video and audio calls	Qualitative content analysis
DeGroot et al. (27)	USA	*N* = 31; mean age 76.3; advanced heart failure and multiple chronic conditions	Convoy-Pal digital palliative care program	Feasibility randomized controlled trial
Medina-García et al. (18)	Spain	Median age 75; median 8 chronic diseases	TeNDER self-care and monitoring tool	Cross-sectional usability study
Sien et al. (20)	Canada	*N* = 18; age 70–80; cancer and arthritis	Tailored symptom-management app prototype	Iterative co-design study
Zhang et al. (32)	China	*N* = 14; mean age 78.6; mean 3.8 conditions and 6.8 medications	Mobile health apps with AI-assisted medication management	Qualitative descriptive study

### 3.3 Types and characteristics of digital health interventions

The included studies implemented a diverse range of digital health interventions targeting older adults with multimorbidity. Based on their functional characteristics and technological features, these interventions were categorized into five main types: (1) integrated digital self-management platforms or mobile health applications, (2) telehealth and remote care management, (3) patient portals and online health services, (4) functional digital support tools, and (5) electronic patient-reported outcome (ePRO) systems (Supplementary Table S4). The categories were developed inductively from the primary function and delivery mode reported for each intervention. Each study was assigned to one mutually exclusive primary category, although individual interventions could include multiple cross-cutting digital functions and delivery or implementation support elements. The categories are presented as pragmatic descriptive groups rather than a one-to-one application of the World Health Organization taxonomy (15). These labels describe core functions and delivery or implementation support elements rather than independently effective components. Across the five categories, the reported descriptors were summarized as core digital functions and delivery or implementation support elements; they were not interpreted as effectiveness-tested components.

Integrated digital self-management platforms and mobile health applications comprised 6/20 studies (30.0%). These interventions typically integrated multiple functions, including symptom monitoring, health education, lifestyle management, and medication reminders. They were intended to support long-term self-management in patients with multimorbidity. For example, Sheng et al. (16) developed the ProACT platform to monitor symptoms and overall health status, while Scheibe et al. (17), Medina-García et al. (18), Nambisan et al. (19), and Sien et al. (20) reported mobile or web-based tools that supported remote monitoring, interactive self-care, personalized tracking, or tailored symptom management. Ha and Park (21) examined the acceptability of ICT-based healthcare and wearable monitoring among older adults with multimorbidity.

Telehealth and remote care management interventions also comprised 6/20 studies (30.0%). They delivered continuous monitoring and professional support through remote physiological tracking, video consultations, or online case management. Many studies used home-based telemonitoring devices or remote rehabilitation models to track physiological indicators, such as blood pressure, blood glucose, or respiratory function. For instance, Wakefield et al. (22) implemented a nurse-managed home telehealth program transmitting daily physiological data; Schmidt et al. (23) conducted case management through video conferencing using tablets; Bernocchi et al. (24) provided home-based telerehabilitation; Donesky et al. (25) delivered remote yoga instruction via television; Buawangpong et al. (26) provided healthcare consultation through video and audio communication; and DeGroot et al. (27) implemented a digital palliative care program.

Patient portals and online health services comprised 3/20 studies (15.0%) and focused on improving communication between patients and healthcare providers and facilitating access to services. Portz et al. (28) examined patient portals for secure messaging, pharmacy management, and laboratory results, while Xu et al. (29) reported the use of online appointment scheduling, teleconsultation services, and social media platforms to support health management. Hall et al. (30) described technology-supported self-management and health-information seeking among older adults with cancer and multimorbidity.

Functional digital support tools comprised 3/20 studies (15.0%) and targeted specific needs in multimorbidity management. Examples included virtual pillbox applications such as ALICE (31) to reduce medication confusion, AI-based home medication optimization tools (32), and photo diary systems for disease management (33).

Electronic patient-reported outcome (ePRO) systems comprised 2/20 studies (10.0%) and collected patient-reported symptoms, goal attainment, and health status periodically through digital platforms. Steele Gray et al. (34) used an ePRO system to monitor goal progress, while Tahsin et al. (35) gathered goal-oriented care data via mobile applications and web portals, providing real-time support for clinical assessment and personalized care planning.

Overall, existing digital health interventions primarily focused on supporting patient self-management, enabling remote health monitoring, and facilitating communication between patients and healthcare providers. Considerable variation remained across interventions in terms of functional integration, technological complexity, and adaptability to the complex care needs of older adults with multimorbidity.

### 3.4 Evidence matrix of implementation factors

Implementation-related barriers and facilitators were mapped across five pragmatic domains: individual, technological, care delivery, social, and health system factors.At the study level, a study was counted once within a domain when it reported at least one barrier or facilitator meeting the operational definition for that domain. Studies and factors could contribute to more than one domain; domain-level frequencies were therefore overlapping and were not summed.

Study-level counts were individual factors in 16/20 studies (80.0%), technological factors in 15/20 (75.0%), care delivery factors in 11/20 (55.0%), social factors in 7/20 (35.0%), and health system factors in 4/20 (20.0%). These domain-level frequencies provide an evidence map rather than estimates of the relative importance or effect of individual determinants. A detailed matrix is presented in Supplementary Table S3.

### 3.5 Thematic synthesis of implementation barriers and facilitators

To provide a more structured interpretation, the Consolidated Framework for Implementation Research (CFIR) was used as a sensitizing framework during thematic synthesis. The five pragmatic reporting domains were retained to summarize the extracted data and were not intended to correspond one-to-one with CFIR constructs (36).

#### 3.5.1 Individual-level factors

Barriers at the individual level included limited digital literacy, low confidence in using technology, and technology-related anxiety, which hindered engagement with digital health interventions (16–18, 27, 35). Age-related declines, such as visual and hearing impairments or reduced fine motor skills, further challenged usability, particularly for applications with small fonts, complex navigation, or multi-step processes (22, 24). Cognitive changes and poorer overall health also limited access to digital platforms, especially for individuals with multiple chronic conditions (27, 34). The complexity of managing multiple conditions increased the burden of self-management tasks, including symptom monitoring, medication adherence, and health record tracking (27, 31, 32). Facilitators included higher self-efficacy, perceived usefulness of digital tools, and structured training programs that enhanced digital literacy and confidence (28, 34).

#### 3.5.2 Technological factors

Technological factors were frequently reported in relation to implementation. Barriers included digital tools not specifically designed for older users, leading to complex interfaces, small text displays, and excessive functional layers that increased the learning burden and reduced usability (27). Limited interoperability between systems hindered effective data sharing and continuity of care (34). System instability and limited internet connectivity further restricted the use of telehealth and mobile health applications (26, 27). Privacy and data security concerns were commonly reported, as older adults worried about misuse or leakage of personal health information (27, 34). Facilitators included simplified user interfaces, larger text displays, voice-assisted guidance, automated data collection, and user-centered design approaches involving older adults in the development process (19, 27, 31).

#### 3.5.3 Care delivery factors

Barriers in care delivery included limited digital training among healthcare professionals, low experience with digital tools, and increased workload associated with monitoring interventions (22–25). Facilitators comprised structured clinical guidance, regular remote follow-up through digital platforms, and coordinated care models involving healthcare professionals and family caregivers (26, 27). Family members and informal caregivers were reported as providing practical support with device operation, health monitoring, and continued engagement (35).

#### 3.5.4 System and social factors

Barriers at the system and social levels included limited integration into healthcare systems, inadequate infrastructure, restricted access to digital devices, and socioeconomic disparities contributing to the digital divide (27, 28). Privacy and security concerns also affected trust in digital health services (34). Facilitators included institutional support, improved infrastructure, integration of digital services into routine care, community-based digital literacy initiatives, and social support from family or community organizations (16–18, 27).

Table 2 provides a structured overview of key barriers and facilitators across these domains. The pragmatic five-domain organization, interpreted with CFIR as a sensitizing framework, helps situate these factors within the broader implementation process and may inform future intervention design and policy development.

**Table 2:** Summary of barriers and facilitators across implementation domains.

Domain	Barriers	Facilitators
Individual factors	Limited digital literacy or operational experience Age-related sensory, cognitive, or fine-motor limitations Technology anxiety or low self-efficacy High self-management workload	Perceived usefulness of digital tools Skills and confidence gained through structured training Motivation to support self-management and independence
Technological factors	Complex or insufficiently age-friendly interfaces System instability, slow responses, or alert failures Limited compatibility or interoperability across devices Unstable internet connection or device/connectivity requirements	Age-friendly interfaces, including larger fonts, simplified workflows, and voice prompts User-centered co-design and tailoring Reliable, functional platforms
Care delivery factors	Limited access to tailored training and professional support Additional clinical workload associated with digital monitoring Limited integration of digital tools into routine care pathways	Ongoing guidance and feedback from healthcare providers Case coordination and multidisciplinary collaboration Coordinated support from healthcare professionals, families, and communities
Health system factors	Administrative or bureaucratic barriers Inadequate enabling resources or infrastructure Unequal distribution of digital resources and network coverage	Institutional support and enabling resources Integration of digital services within routine care systems Improved infrastructure and network coverage
Social factors	Privacy and data-security concerns affecting trust Digital divide related to education, income, or access to devices Limited trust in online healthcare services	Practical support from family members, caregivers, and peers Peer communication and supportive social networks Community-based digital literacy support

Domains are pragmatic summaries of reported factors. A factor could contribute to more than one domain; categories are not mutually exclusive and do not indicate relative importance.

## 4 Discussion

### 4.1 Key findings

This scoping review synthesized evidence on the barriers and facilitators influencing the implementation of digital health interventions (DHIs) among older adults with multimorbidity. Overall, the findings indicate that, although DHIs are increasingly adopted to support chronic disease management, their implementation remains influenced by multiple interacting determinants, including individual capabilities, technological usability, caregiver support, and broader healthcare system context. These results underscore the inherent complexity of implementing digital health technologies among populations with multiple chronic conditions and age-related functional limitations.

Compared with recent reviews of digital health in broader populations of older adults with chronic conditions, which have largely focused on clinical outcomes or determinants of technology adoption, this review examines implementation in the context of multimorbidity and continuing care (5, 7). It brings together intervention types and functions, reported implementation determinants, and delivery or implementation support elements. Across the included studies, digital health interventions were used for integrated self-management, remote care management, access to online services, task-specific support, and electronic patient-reported outcome monitoring. These varied applications suggest that implementation is shaped not only by the technology itself, but also by how its functions are incorporated into ongoing multimorbidity care. Related reviews have addressed digital technologies for multimorbidity management, self-management applications, and hospital-to-home transitions in older populations, although their target populations and review questions differ (10–12). The present review therefore complements this literature by examining implementation in continuing care for older adults with multimorbidity and mapping intervention functions, implementation determinants, and delivery or implementation support elements together.

Implementation-related factors were identified across all five pragmatic domains. The study-level counts presented in the evidence matrix are descriptive and non-mutually exclusive; they map the distribution of reported factors across studies rather than quantify the relative importance of individual, technological, care-delivery, social, or health-system determinants. The included studies also reported support elements, such as age-friendly design, structured training, technical assistance, professional follow-up, caregiver involvement, care coordination, and organizational support, that may be useful when planning implementation. Taken together, the findings show how intervention types and functions intersect with reported implementation determinants and delivery or implementation support elements relevant to adoption, integration into care, continued use, and sustainability. As this was a scoping review, these mapped patterns should not be interpreted as estimates of the relative effects or importance of individual factors.

The findings can be interpreted through a socioecological perspective, which conceptualizes health behaviors and intervention outcomes as shaped by interacting influences at individual, interpersonal, organizational, community, and policy levels (37). In this context, individual-level factors relate to older adults' functional capacity, digital skills, and health literacy (27, 34). Interpersonal influences reflect the support of family caregivers and healthcare professionals (27, 35). Organizational factors concern the integration of digital technologies into healthcare services, while community and system-level conditions, such as digital infrastructure and socioeconomic resources, shape the accessibility and sustainability of DHIs (28, 29). The socioecological perspective, treatment burden, and the digital divide are used only as explanatory perspectives in the discussion; they did not inform the search strategy, eligibility criteria, data extraction, or primary classification. In relation to the review questions, the findings map intervention types and functions, implementation barriers and facilitators, and reported delivery or implementation support elements; they also identify implications for implementation planning in continuing care, including community and public health nursing practice. These observations describe reported implementation experiences and do not establish the relative effectiveness of any individual function or support element.

### 4.2 Treatment burden and functional capacity

A key mechanism underlying many of the implementation challenges identified in this review is the imbalance between treatment workload and patients' functional capacity. Burden of treatment refers to the work that healthcare demands place on patients and the consequences of that work for daily functioning (38). Older adults with multimorbidity frequently manage multiple medications, symptom monitoring, and repeated healthcare encounters, all of which contribute to substantial self-management demands (27, 31, 34).

Although DHIs are designed to support chronic disease management, poorly designed digital interventions may unintentionally increase treatment workload (39). For example, digital systems that require frequent log-ins, manual data entry, or the simultaneous use of multiple devices may increase management complexity. Such demands may exceed older adults' cognitive, sensory, or physical capacities, ultimately reducing engagement with digital health tools.

Therefore, the design of DHIs should prioritize usability and minimize additional workload for patients. Simplifying user interfaces, incorporating automated data collection, and integrating digital tools within existing healthcare systems may help reduce treatment burden and improve the sustainability of digital health interventions for older adults with multimorbidity (40).

### 4.3 Social and contextual influences

Beyond individual and technological factors, the implementation of DHIs is also shaped by broader social and contextual environments. Limited internet access, inadequate digital literacy, economic constraints, and difficulty navigating digital health services may restrict older adults' engagement with digital technologies (41–43). These structural barriers are often conceptualized within the digital divide, which encompasses disparities in access to digital resources and digital health services (41).

Family caregivers and healthcare professionals were reported as potential sources of practical support in addressing these gaps. For many older adults, family members provide essential support in operating devices, navigating digital platforms, and reporting health data (44). Similarly, ongoing professional guidance from healthcare providers can enhance patients' confidence in using digital technologies and support long-term adherence to digital health interventions (45).

These findings suggest that digital health implementation should not be considered solely a technological issue. Rather, it represents a socially embedded process that depends on supportive interpersonal relationships, accessible community resources, and healthcare system integration.

### 4.4 Implications for public health nursing practice

The findings of this review suggest that public health nurses, alongside other community and primary care professionals, may contribute to the implementation of digital health interventions within continuing-care settings. Their potential contribution includes supporting older adults in navigating digital health technologies while addressing complex chronic disease management needs.

During routine community visits or follow-up care, nurses may assess patients' digital literacy, functional capacity, and treatment burden and, within their roles and available resources, provide individualized guidance to facilitate the use of digital health tools. Integrating remotely collected health data into routine nursing assessments may also support earlier recognition of health deterioration and communication among patients, family caregivers, and healthcare providers (46, 47).

From a public health nursing perspective, addressing the barriers identified in this review requires strategies that operate across multiple ecological levels. These may include strengthening individual digital literacy, supporting family caregiving systems, integrating digital technologies within community health services, and promoting equitable access to digital health resources. Such efforts may help ensure that digital health innovations contribute to more equitable and person-centered chronic disease management for older adults with multimorbidity.

### 4.5 Limitations and future research

Several limitations should be acknowledged. First, the review was restricted to peer-reviewed studies published in English or Chinese and may therefore be subject to language and publication bias. Gray literature, including service evaluations, policy documents, and institutional reports, was not searched and may contain additional implementation-relevant information. Second, as this was a scoping review, no formal methodological quality appraisal was undertaken. The included studies also varied substantially in design, intervention type, and implementation context, which limits direct comparison across the evidence base.

The review was intentionally focused on interventions with a continuing-care function and does not represent digital health interventions confined to an acute inpatient episode. In addition, caregivers and healthcare professionals were treated as implementation stakeholders rather than independent target populations; studies reporting only their perspectives may therefore have been underrepresented. Finally, the allocation of findings across thematic domains involved reviewer judgment, and multi-level implementation factors could reasonably overlap across domains. These considerations should be taken into account when interpreting the reported distribution of implementation determinants and delivery or implementation support elements.

Future research should further evaluate implementation outcomes, including adoption, integration into care, reach, continued use, scalability, and long-term sustainability, alongside effectiveness across continuing-care settings. In particular, applying implementation science frameworks may provide valuable insights into strategies for integrating digital health technologies into routine chronic disease management for older adults with multimorbidity.

## 5 Conclusion

This scoping review provides a systematic summary of the factors influencing the implementation of digital health interventions for older adults with multimorbidity. Our findings suggest that, while digital health technologies have potential to support chronic disease self-management, their implementation and sustained use are shaped by a complex interplay of multi-level factors, including individual capabilities, technical usability, care support, and the broader healthcare system environment. Several included studies reported limitations in integration and age-friendly design, which may impede longer-term use and wider uptake.

Future design and implementation of digital health interventions should prioritize patient-centered, age-friendly design while enhancing sustainability through interdisciplinary collaboration and continuing-care support. Public health nurses and other members of multidisciplinary care teams may contribute to implementation by supporting digital literacy, coordinating care resources, and helping to integrate digital tools into routine services. Strengthening implementation support across community and continuing-care settings may help improve the accessibility and usability of digital health services for older adults living with multimorbidity.

## Data Availability

The original contributions presented in the study are included in the article/Supplementary material, further inquiries can be directed to the corresponding author.
