# Supplementary material for: Implementation barriers and facilitators of digital health interventions for older adults with multimorbidity: a scoping review
Source: Front Public Health. 2026 Jul 28;14:1896061. doi: 10.3389/fpubh.2026.1896061 (PMC13457100; doi:10.3389/fpubh.2026.1896061)
Supplement: Supplementary file 1 [file Table_1.docx]

Supplementary Material

**Supplementary Table S1. Full search strategies.**

Note. Searches were conducted on 23 June 2026, from the earliest searchable or indexed date available in each database to the search date. Search strategies are presented separately by database and were adapted to database-specific syntax and indexing characteristics. In Web of Science Core Collection, PubMed, CINAHL, and Embase, the search combined multimorbidity, older adults, and digital health interventions. To maximize sensitivity in CNKI and Wanfang, the electronic query combined multimorbidity and digital health intervention terms, and the age eligibility criterion was applied during screening. Implementation-related terms were not used as mandatory search blocks. English- or Chinese-language peer-reviewed empirical studies were eligible for inclusion. Language and publication-type eligibility criteria were applied during screening; implementation relevance was assessed at title and abstract screening and confirmed at full-text review.

| **Search step** | **Search query** | **Records retrieved** |
| --- | --- | --- |
| 1 | TS=( multimorbid* OR "multi-morbid*" OR polymorbid* OR plurimorbid* OR "multiple morbidity" OR "multiple morbidities" OR "multiple chronic condition*" OR "multiple chronic disease*" OR "multiple chronic illness*" OR "multiple long-term condition*" OR "multiple long term condition*" OR comorbid* ) | 359947 |
| 2 | TS=( "older adult*" OR "older person*" OR "older people" OR "older patient*" OR elderly OR elder* OR geriatric* OR "senior citizen*" ) | 806938 |
| 3 | TS=( "digital health" OR ehealth OR "e-health" OR mhealth OR "m-health" OR telehealth OR telemedicine OR telecare OR "tele-care" OR telemonitor* OR "remote monitor*" OR "remote patient monitor*" OR "remote consultation*" OR teleconsult* OR "remote care" OR "virtual care" OR "virtual health*" OR "video consultation*" OR "video visit*" OR videoconferenc* OR telerehab* OR "tele-rehabilitation" OR "remote rehabilitation" OR "mobile health" OR "mobile app*" OR "health app*" OR "mobile application*" OR smartphone* OR "smart phone*" OR "web-based" OR "web based" OR webbased OR "internet-based" OR "internet based" OR "online health*" OR "online intervention*" OR "digital intervention*" OR "digital therapeutic*" OR "digital self-management" OR "digital self management" OR "wearable device*" OR "wearable technolog*" OR smartwatch* OR "activity tracker*" OR "fitness tracker*" OR "patient portal*" OR "personal health record*" OR ePRO OR "electronic patient-reported outcome*" OR "electronic patient reported outcome*" OR "text messag*" OR "short message service" OR "interactive voice response" OR chatbot* OR "conversational agent*" OR "digital care coordination" OR "digital care-coordination" OR "care coordination platform*" ) | 485379 |
| 4 | #1 AND #2 AND #3 | 903 |

**Web of Science Core Collection**

**PubMed**

| **Search step** | **Search query** | **Records retrieved** |
| --- | --- | --- |
| 1 | ( "Multimorbidity"[Mesh] OR multimorbid*[tiab] OR multi-morbid*[tiab] OR polymorbid*[tiab] OR plurimorbid*[tiab] OR "multiple morbidity"[tiab] OR "multiple morbidities"[tiab] OR "multiple chronic condition"[tiab] OR "multiple chronic conditions"[tiab] OR "multiple chronic disease"[tiab] OR "multiple chronic diseases"[tiab] OR "multiple chronic illness"[tiab] OR "multiple chronic illnesses"[tiab] OR "multiple long-term condition"[tiab] OR "multiple long-term conditions"[tiab] OR "multiple long term condition"[tiab] OR "multiple long term conditions"[tiab] OR comorbid*[tiab] ) | 361287 |
| 2 | ( "Aged"[Mesh] OR "older adult"[tiab] OR "older adults"[tiab] OR "older person"[tiab] OR "older persons"[tiab] OR "older people"[tiab] OR "older patient"[tiab] OR "older patients"[tiab] OR elderly[tiab] OR elder*[tiab] OR geriatric*[tiab] OR "senior citizen"[tiab] OR "senior citizens"[tiab] ) | 4098999 |
| 3 | ( "Digital Health"[Mesh] OR "Telemedicine"[Mesh] OR "Remote Consultation"[Mesh] OR "Remote Patient Monitoring"[Mesh] OR "Telerehabilitation"[Mesh] OR "Mobile Applications"[Mesh] OR "Wearable Electronic Devices"[Mesh] OR "Patient Portals"[Mesh] OR "Health Records, Personal"[Mesh] OR "Text Messaging"[Mesh] OR "Videoconferencing"[Mesh] OR "digital health"[tiab] OR ehealth[tiab] OR "e-health"[tiab] OR mhealth[tiab] OR "m-health"[tiab] OR telehealth[tiab] OR telemedicine[tiab] OR telecare[tiab] OR telemonitor*[tiab] OR "remote monitoring"[tiab] OR "remote patient monitoring"[tiab] OR "remote consultation"[tiab] OR teleconsult*[tiab] OR "remote care"[tiab] OR "virtual care"[tiab] OR "video consultation"[tiab] OR "video consultations"[tiab] OR "video visit"[tiab] OR "video visits"[tiab] OR videoconferenc*[tiab] OR telerehab*[tiab] OR "tele-rehabilitation"[tiab] OR "remote rehabilitation"[tiab] OR "mobile health"[tiab] OR "mobile app"[tiab] OR "mobile apps"[tiab] OR "health app"[tiab] OR "health apps"[tiab] OR "mobile application"[tiab] OR "mobile applications"[tiab] OR smartphone*[tiab] OR "smart phone"[tiab] OR "smart phones"[tiab] OR "web-based"[tiab] OR "web based"[tiab] OR "internet-based"[tiab] OR "internet based"[tiab] OR "online health"[tiab] OR "online intervention"[tiab] OR "online interventions"[tiab] OR "digital intervention"[tiab] OR "digital interventions"[tiab] OR "digital therapeutic"[tiab] OR "digital therapeutics"[tiab] OR "digital self-management"[tiab] OR "digital self management"[tiab] OR "wearable device"[tiab] OR "wearable devices"[tiab] OR smartwatch*[tiab] OR "activity tracker"[tiab] OR "activity trackers"[tiab] OR "fitness tracker"[tiab] OR "fitness trackers"[tiab] OR "patient portal"[tiab] OR "patient portals"[tiab] OR "personal health record"[tiab] OR "personal health records"[tiab] OR ePRO[tiab] OR "electronic patient-reported outcome"[tiab] OR "electronic patient-reported outcomes"[tiab] OR "text messaging"[tiab] OR "short message service"[tiab] OR "interactive voice response"[tiab] OR chatbot*[tiab] OR "conversational agent"[tiab] OR "conversational agents"[tiab] ) | 278740 |
| 4 | #1 AND #2 AND #3 | 1723 |

**CINAHL**

| **Search step** | **Search query** | **Records retrieved** |
| --- | --- | --- |
| S1 | ( MH "Multimorbidity+" OR TX ( multimorbid* OR "multi-morbid*" OR polymorbid* OR plurimorbid* OR "multiple morbidity" OR "multiple morbidities" OR "multiple chronic condition*" OR "multiple chronic disease*" OR "multiple chronic illness*" OR "multiple long-term condition*" OR "multiple long term condition*" OR comorbid* ) ) | 752985 |
| S2 | ( MH "Aged+" OR TX ( "older adult*" OR "older person*" OR "older people" OR "older patient*" OR elderly OR elder* OR geriatric* OR "senior citizen*" ) ) | 2798503 |
| S3 | ( MH "Telemedicine+" OR MH "Mobile Applications" OR MH "Wearable Electronic Devices" OR MH "Patient Portals" OR MH "Personal Health Records" OR MH "Text Messaging" OR MH "Videoconferencing" OR TX ( "digital health" OR ehealth OR "e-health" OR mhealth OR "m-health" OR telehealth OR telemedicine OR telecare OR "tele-care" OR telemonitor* OR "remote monitor*" OR "remote patient monitor*" OR "remote consultation*" OR teleconsult* OR "remote care" OR "virtual care" OR "virtual health*" OR "video consultation*" OR "video visit*" OR videoconferenc* OR telerehab* OR "tele-rehabilitation" OR "remote rehabilitation" OR "mobile health" OR "mobile app*" OR "health app*" OR "mobile application*" OR smartphone* OR "smart phone*" OR "web-based" OR "web based" OR webbased OR "internet-based" OR "internet based" OR "online health*" OR "online intervention*" OR "digital intervention*" OR "digital therapeutic*" OR "digital self-management" OR "digital self management" OR "wearable device*" OR "wearable technolog*" OR smartwatch* OR "activity tracker*" OR "fitness tracker*" OR "patient portal*" OR "personal health record*" OR ePRO OR "electronic patient-reported outcome*" OR "electronic patient reported outcome*" OR "text messag*" OR "short message service" OR "interactive voice response" OR chatbot* OR "conversational agent*" OR "digital care coordination" OR "digital care-coordination" OR "care coordination platform*" ) ) | 1059013 |
| S4 | S1 AND S2 AND S3 | 2154 |

**Embase**

| **Search step** | **Search query** | **Records retrieved** |
| --- | --- | --- |
| S1 | ('multimorbidity'/exp OR multimorbid*:ti,ab OR 'multi-morbid*':ti,ab OR polymorbid*:ti,ab OR plurimorbid*:ti,ab OR 'multiple morbidity':ti,ab OR 'multiple morbidities':ti,ab OR 'multiple chronic condition*':ti,ab OR 'multiple chronic disease*':ti,ab OR 'multiple chronic illness*':ti,ab OR 'multiple long-term condition*':ti,ab OR 'multiple long term condition*':ti,ab) | 29437 |
| S2 | 'aged'/exp OR 'older adult*':ti,ab OR 'older person*':ti,ab OR 'older people':ti,ab OR 'older patient*':ti,ab OR elderly:ti,ab OR elder*:ti,ab OR geriatric*:ti,ab OR 'senior citizen*':ti,ab | 5099159 |
| S3 | 'digital health'/exp OR 'telemedicine'/exp OR 'remote consultation'/exp OR 'remote patient monitoring'/exp OR 'telerehabilitation'/exp OR 'mobile application'/exp OR 'wearable device'/exp OR 'patient portal'/exp OR 'personal health record'/exp OR 'text messaging'/exp OR 'videoconferencing'/exp OR 'digital health':ti,ab OR ehealth:ti,ab OR 'e-health':ti,ab OR mhealth:ti,ab OR 'm-health':ti,ab OR telehealth:ti,ab OR telemedicine:ti,ab OR telecare:ti,ab OR 'tele-care':ti,ab OR telemonitor*:ti,ab OR 'remote monitor*':ti,ab OR 'remote patient monitor*':ti,ab OR 'remote consultation*':ti,ab OR teleconsult*:ti,ab OR 'remote care':ti,ab OR 'virtual care':ti,ab OR 'virtual health*':ti,ab OR 'video consultation*':ti,ab OR 'video visit*':ti,ab OR videoconferenc*:ti,ab OR telerehab*:ti,ab OR 'tele-rehabilitation':ti,ab OR 'remote rehabilitation':ti,ab OR 'mobile health':ti,ab OR 'mobile app*':ti,ab OR 'health app*':ti,ab OR 'mobile application*':ti,ab OR smartphone*:ti,ab OR 'smart phone*':ti,ab OR 'web-based':ti,ab OR 'web based':ti,ab OR webbased:ti,ab OR 'internet-based':ti,ab OR 'internet based':ti,ab OR 'online health*':ti,ab OR 'online intervention*':ti,ab OR 'digital intervention*':ti,ab OR 'digital therapeutic*':ti,ab OR 'digital self-management':ti,ab OR 'digital self management':ti,ab OR 'wearable device*':ti,ab OR 'wearable technolog*':ti,ab OR smartwatch*:ti,ab OR 'activity tracker*':ti,ab OR 'fitness tracker*':ti,ab OR 'patient portal*':ti,ab OR 'personal health record*':ti,ab OR epro:ti,ab OR 'electronic patient-reported outcome*':ti,ab OR 'electronic patient reported outcome*':ti,ab OR 'text messag*':ti,ab OR 'short message service':ti,ab OR 'interactive voice response':ti,ab OR chatbot*:ti,ab OR 'conversational agent*':ti,ab OR 'digital care coordination':ti,ab OR 'digital care-coordination':ti,ab OR 'care coordination platform*':ti,ab | 830868 |
| S4 | #1 AND #2 AND #3 | 1600 |

**CNKI**

| **Search step** | **Search query** | **Records retrieved** |
| --- | --- | --- |
| S1 | SU=(多病共存+多重慢性病+多种慢性病+多疾病共存+慢性病共病+慢性病共存+多重共病) AND SU=(数字健康+数字医疗+互联网医疗+移动健康+远程医疗+远程监测+远程护理+远程随访+远程康复+可穿戴设备+健康APP+智慧医疗) | 16 |

**Wanfang**

| **Search step** | **Search query** | **Records retrieved** |
| --- | --- | --- |
| S1 | 主题:(多病共存 OR 多重慢性病 OR 多种慢性病 OR 多种慢性疾病OR 多疾病共存 OR 慢性病共病 OR 慢性病共存 OR 多重共病)AND(数字健康 OR 数字医疗 OR 数字卫生 OR 电子健康OR 互联网医疗 OR 移动健康 OR 移动医疗OR 远程医疗 OR 远程健康 OR 远程照护 OR 远程护理OR 远程监测 OR 远程随访 OR 远程会诊 OR 远程康复OR 可穿戴设备 OR 可穿戴技术 OR 智能穿戴OR 健康APP OR 健康应用 OR 移动应用 OR 手机应用 OR 智能手机应用OR 数字疗法 OR 数字化健康管理 OR 数字化自我管理OR 健康管理平台 OR 在线健康服务 OR 线上健康服务OR 视频问诊 OR 短信干预 OR 智慧医疗 OR 智慧健康) | 541 |

**Supplementary Table S2. PRISMA-ScR checklist.**

| **Item** | **Section/topic** | **Checklist item** | **Location in manuscript** |
| --- | --- | --- | --- |
| 1 | Title | Identify the report as a scoping review. | Title page |
| 2 | Abstract | Provide a structured summary including background, objectives, eligibility criteria, sources of evidence, charting methods, results, and conclusions. | Abstract |
| 3 | Rationale | Describe the rationale for the review in the context of existing knowledge. | Introduction |
| 4 | Objectives | Provide an explicit statement of the questions and objectives being addressed. | Review questions |
| 5 | Protocol and registration | Indicate whether a review protocol exists and where it can be accessed. | Methods, Study design (a review protocol was not registered). |
| 6 | Eligibility criteria | Specify characteristics of sources of evidence used as eligibility criteria and provide a rationale. | Eligibility criteria |
| 7 | Information sources | Describe all information sources and the date of the most recent search. | Search strategy; Supplementary Table S1 |
| 8 | Search | Present the full electronic search strategy for at least one database. | Supplementary Table S1 |
| 9 | Selection of sources of evidence | State the process for selecting sources of evidence. | Study selection |
| 10 | Data charting process | Describe methods of charting data and whether forms were piloted. | Data extraction (a standardized data-charting form was used; formal pilot testing was not reported). |
| 11 | Data items | List and define all variables for which data were sought. | Data extraction |
| 12 | Critical appraisal of individual sources of evidence | If done, provide rationale and methods for critical appraisal. | Not applicable; critical appraisal was not conducted because this was a scoping review. |
| 13 | Synthesis of results | Describe methods of handling and summarizing data. | Data synthesis |
| 14 | Selection of sources of evidence | Give numbers of sources screened, assessed for eligibility, and included, with reasons for exclusion. | Results; Figure 1 |
| 15 | Characteristics of sources of evidence | Present characteristics of each source of evidence. | Table 1 |
| 16 | Critical appraisal within sources of evidence | If done, present critical appraisal results. | Not applicable; critical appraisal was not conducted because this was a scoping review. |
| 17 | Results of individual sources of evidence | Present relevant data charted from included sources. | Results; Table 1; Supplementary Tables S3-S4 |
| 18 | Synthesis of results | Summarize results in relation to review questions. | Results; Table 2 |
| 19 | Summary of evidence | Summarize main results and link them to review questions and objectives. | Discussion |
| 20 | Limitations | Discuss limitations of the scoping review process. | Limitations and future research |
| 21 | Conclusions | Provide a general interpretation of the results and implications. | Conclusion |
| 22 | Funding | Describe sources of funding and role of funders. | Review funding reported; funder role and included-source funding not reported. |

**Supplementary Table S3. Evidence matrix of implementation factors.**

| Author (year) | Individual factors (I) | Technological factors (T) | Care delivery factors (C) | Health system factors (Sy) | Social factors (So) |
| --- | --- | --- | --- | --- | --- |
| Wakefield (2011) | - | B: landline requirements | - | - | - |
| Mira (2014) | F: independence | B: alert failures | F: intensive training | - | - |
| Donesky (2017) | F: interest in participation | B: screen lag; outdated hardware | - | - | - |
| Bernocchi (2018) | - | - | F: multidisciplinary collaboration | - | - |
| Portz (2019) | B: anxiety | B: interface design; software upgrades | F: patient-provider communication | - | F: caregiver/family support |
| Schmidt (2019) | F: self-reliance | B: poor rural internet | F: expert support | B: bureaucratic paperwork | F: reduced isolation |
| Ha (2020) | B: technology anxiety; F: perceived usefulness | - | - | F: facilitating resources | - |
| Scheibe (2021) | B: initial insecurity; F: perceived security | B: hardware robustness issues | F: personal support | - | - |
| Hall (2021) | F: user confidence | B: medical terminology difficulties | B: fear of clinician judgment | - | F: peer communication |
| Steele Gray (2021) | F: perceived meaningfulness | F: co-design; B: system complexity; design complexity | - | - | - |
| Kuo (2022) | B: lack of confidence; limited operational skills | - | - | - | - |
| Tahsin (2022) | F: self-efficacy | B: login issues | B: clinician workload; F: provider relationships | - | - |
| Sheng (2022) | - | B: syncing issues | - | - | - |
| Nambisan (2023) | - | - | F: clinician input | - | B: privacy/security concerns |
| Xu (2023) | B: cognitive burden; F: perceived convenience | B: software problems; unstable internet connection | - | B: infrastructure limitations | - |
| Buawangpong (2024) | B: sensory decline | - | - | B: infrastructure limitations | B: dependence on family support |
| DeGroot (2024) | B: disease complexity | B: technical frustration | F: palliative care support | - | - |
| Medina-Garcia (2024) | F: higher education level | B: low efficiency | - | - | B: need for assistance |
| Sien (2024) | F: fit with routine | B: browser/native app limitations | F: patient-provider communication | - | - |
| Zhang (2025) | B: traditional beliefs | B: lack of age-friendly design | B: feedback disconnection | - | B: economic burden; technology gap |

Note. At the study level, a study was counted once within a domain when it reported at least one barrier or facilitator meeting the operational definition for that domain. Studies and factors could contribute to more than one domain; domain-level frequencies were therefore overlapping and were not summed.

**Supplementary Table S4. Types and characteristics of digital health interventions. This table also summarizes cross-cutting core digital functions and delivery or implementation support elements, with WHO correspondence shown as a conceptual, non-exclusive comparison.**

| **Primary intervention category** | **Representative studies** | **Main functions/intervention content** | **Technical features and multimorbidity fit** |
| --- | --- | --- | --- |
| Integrated self-management platforms and mobile health applications | Sheng (2022) [ProACT]; Scheibe (2021) [Motiva]; Medina-Garcia (2024) [TeNDER]; Nambisan (2023) [myHESTIA]; Sien (2024) [tailored symptom-management app]; Ha (2020) [ICT/wearable acceptance] | Integrated symptom monitoring, health indicators, medication support, personalized education, tracking, alerts, and feedback. | Provides an integrated interface for multiple indicators and long-term self-management functions. |
| Telehealth and remote care management | Wakefield (2011); Schmidt (2019); Donesky (2017); Bernocchi (2018); Buawangpong (2024); DeGroot (2024) | Remote consultations, video conferences, home monitoring, telerehabilitation, palliative care support, and nurse/case-manager follow-up. | Provides remote consultation, monitoring, and follow-up functions, with implementation requirements that may include connectivity, workflow integration, and professional monitoring capacity. |
| Patient portals and online health services | Portz (2019); Xu (2023); Hall (2021) | Electronic record access, secure messaging, pharmacy management, online appointment services, teleconsultation, and online health information seeking. | Provides information-access and communication functions; use may be limited by interface complexity, terminology, trust, and digital literacy. |
| Functional digital support tools | Mira (2014) [ALICE]; Zhang (2025) [AI-assisted medication management]; Kuo (2022) [photo diary] | Medication reminders, visual medication schedules, AI-assisted regimen support, and photo-based disease management reflection. | Provides task-specific support for medication management and daily routines; implementation may require age-friendly design and reliable reminders. |
| Electronic patient-reported outcome systems | Steele Gray (2021); Tahsin (2022) | Digital collection of symptoms, goals, health status, and patient-reported outcomes for clinical assessment and personalized care planning. | Enables digital collection of symptoms, goals, health status, and patient-reported outcomes for clinical assessment and care planning; implementation may increase clinician workload. |
| Cross-cutting analytic descriptors | All five intervention categories | Core digital functions: monitoring; reminders; education; communication; self-management support; data feedback; and symptom or goal reporting. | Delivery or implementation support elements: age-friendly design; personalization; training; technical support; caregiver involvement; professional follow-up; workflow integration; and care coordination. |
| WHO conceptual correspondence | Conceptual comparison only | Broadly relates to client-facing information, communication, reminders, self-management, monitoring, and reporting functions. | May also relate to provider- or system-facing telemedicine, data review, workflow integration, and care coordination. The correspondence is non-exclusive and is not a one-to-one taxonomy mapping. |

Note. The five intervention categories were mutually exclusive primary classifications based on the main functions and delivery modes reported in the included studies: integrated self-management platforms and mobile health applications (6/20, 30.0%), telehealth and remote care management (6/20, 30.0%), patient portals and online health services (3/20, 15.0%), functional digital support tools (3/20, 15.0%), and electronic patient-reported outcome systems (2/20, 10.0%). Individual interventions could nevertheless contain multiple core digital functions and multiple delivery or implementation support elements. Correspondence with the World Health Organization Classification of Digital Interventions, Services and Applications in Health is conceptual rather than a strict one-to-one mapping. Functions and support elements were reported features of the included interventions and were not evaluated as independently effective components.
